# Dual RNA-Seq of *Flavobacterium psychrophilum* and Its Outer Membrane Vesicles Distinguishes Genes Associated with Susceptibility to Bacterial Cold-Water Disease in Rainbow Trout (*Oncorhynchus mykiss*)

**DOI:** 10.3390/pathogens12030436

**Published:** 2023-03-10

**Authors:** Pratima Chapagain, Ali Ali, Mohamed Salem

**Affiliations:** 1 Department of Biology and Molecular Biosciences Program, Middle Tennessee State University, Murfreesboro, TN 37132, USA; 2 Department of Animal and Avian Sciences, University of Maryland, College Park, MD 20742, USA

**Keywords:** cell wall hydrolase, ribosome, translation, DNA-binding proteins, methyltransferases

## Abstract

*Flavobacterium psychrophilum* (*Fp*), the causative agent of Bacterial Cold-Water disease in salmonids, causes substantial losses in aquaculture. Bacterial outer membrane vesicles (OMVs) contain several virulence factors, enzymes, toxins, and nucleic acids and are expected to play an essential role in host–pathogen interactions. In this study, we used transcriptome sequencing, RNA-seq, to investigate the expression abundance of the protein-coding genes in the *Fp* OMVs versus the *Fp* whole cell. RNA-seq identified 2190 transcripts expressed in the whole cell and 2046 transcripts in OMVs. Of them, 168 transcripts were uniquely identified in OMVs, 312 transcripts were expressed only in the whole cell, and 1878 transcripts were shared in the two sets. Functional annotation analysis of the OMV-abundant transcripts showed an association with the bacterial translation machinery and histone-like DNA-binding proteins. RNA-Seq of the pathogen transcriptome on day 5 post-infection of *Fp*-resistant versus *Fp*-susceptible rainbow trout genetic lines revealed differential gene expression of OMV-enriched genes, suggesting a role for the OMVs in shaping the host–microbe interaction. Interestingly, a cell wall-associated hydrolase (CWH) gene was the most highly expressed gene in OMVs and among the top upregulated transcripts in susceptible fish. The CWH sequence was conserved in 51 different strains of *Fp*. The study provides insights into the potential role of OMVs in host–pathogen interactions and explores microbial genes essential for virulence and pathogenesis.

## 1. Introduction

*Flavobacterium psychrophilum* (*Fp*) is a harmful pathogen that causes Bacterial Cold-Water Disease (BCWD) in salmonids [1,2]. *Fp* is a Gram-negative bacterium affecting all species of salmonids, and infections occur worldwide. The high mortality caused by *Fp* is due to the ability of the pathogen to survive in harsh environmental conditions, multiple transmission routes, and the unavailability of effective vaccines [3]. This bacterium can be detected in water samples and can form biofilms on environmental and host surfaces, which aids bacteria in pathogenesis [4]. The bacterium primarily affects fry with an underlying immune system, whereas upon infection in adult fish, necrotic lesions are developed, resulting in hemorrhagic septicemia [5], and because of this, the salmonid aquaculture industries suffer from considerable losses annually.

Several breeding approaches have been used to assist in the genetic improvement of hosts for BCWD resistance [6]. Moreover, some efforts have been made to understand the role of bacterial genes in causing the disease. Previous studies on *Flavobacterium columnare* and *Flavobacterium johnsoniae* identified several peptidase enzymes and cell surface adhesin genes involved in causing the disease [5,7,8]. To some extent, virulence factors, such as extracellular proteases, are associated with host tissue destruction [9]. Similarly, an iron-uptake-associated gene has also been studied as a virulence gene because iron acquisition allows the pathogenic bacteria to scavenge iron and grow inside the host; *Fp* strains that lack these genes are less virulent [10].

Evidence has emerged that the bacterial outer membrane vesicle-mediated delivery of virulence factors, proteins, and nucleic acids embedded inside nanoparticles is a novel mechanism of host–pathogen communication [11,12]. OMVs and their inner content can be transferred to host cells either via endocytosis or attachment to host cell receptors, as proposed for *Pseudomonas aeruginosa* [12]. The presence of proteins and nucleic acids within the prokaryotic OMVs may be explained through the protein synthesis process since OMVs entrap the protein synthesis machinery, including the mRNAs, during vesicle formation. Alternatively, the free-floating inner cargos in the cytoplasm or the cytoplasmic constituents might be entrapped during vesicle formation [13].

The mechanism of cell lysis during cargo transfer to the host has not been explained yet in animals. Studies showed that OMVs contain several enzymes, including cell wall-associated hydrolase (CWH). These hydrolases are enzymes associated with bacterial cell wall degradation and biosynthesis during bacterial growth. They are also involved in cell autolysis and cleavage, which might mediate the transfer of inner cargos to the host cell during the interaction. These enzymes participate in cell lysis during cell division and intercellular communication [14]. In bacteria, cell wall hydrolase enzymes are primarily associated with cell division, cell wall degradation, and biosynthesis during bacterial growth. Bacteriophages use hydrolases to destroy bacterial cell membranes and walls during host–pathogen communication [15].

In this study, we aimed to investigate mRNA transcript abundance in *Fp* OMVs versus *Fp* whole cells, which will help explain the functions of *Fp* genes involved in host–pathogen interactions. A high enrichment of histone-like DNA-binding protein and ribosomal transcripts in OMV was the primary finding of our study. A CWH transcript was the most abundant in OMVs. We also identified protein-coding genes enriched in OMVs and associated their expression with BCWD resistance in two selectively bred rainbow trout genetic lines. The study provides insight into the role of OMVs in the host–pathogen interaction during infection and warrants further physiological and omics studies.

## 2. Results and Discussion

### 2.1. OMV Isolation and TEM

**Figure 1:**
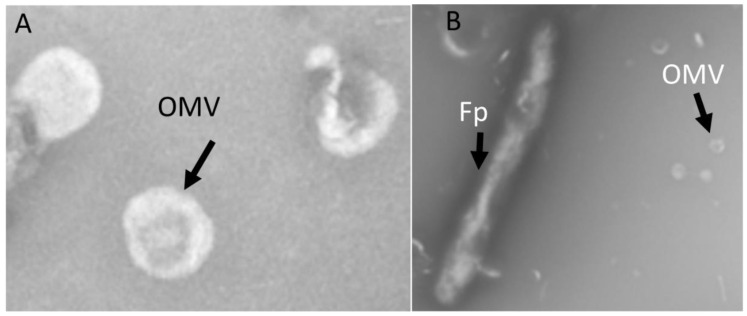
(**A**) Transmission electron microscopy (TEM) of *Fp* OMVs. OMVs appeared as spherical, nano-shaped particles. (**B**) TEM of *Fp*; bacteria appeared rod-shaped with OMVs.

Isolated *Fp* OMVs were observed via TEM as spherical-shaped particles having an average diameter between 50 and 300 nm (Figure 1A), while *Fp* was observed as having a rod-shaped morphology with a size of approximately 2–5 µm. (Figure 1B).

### 2.2. RNA-Seq of Fp Whole Cells and OMVs Identify Protein-Coding Genes Associated with Bacterial Virulence and Pathogenesis

**Figure 2:**
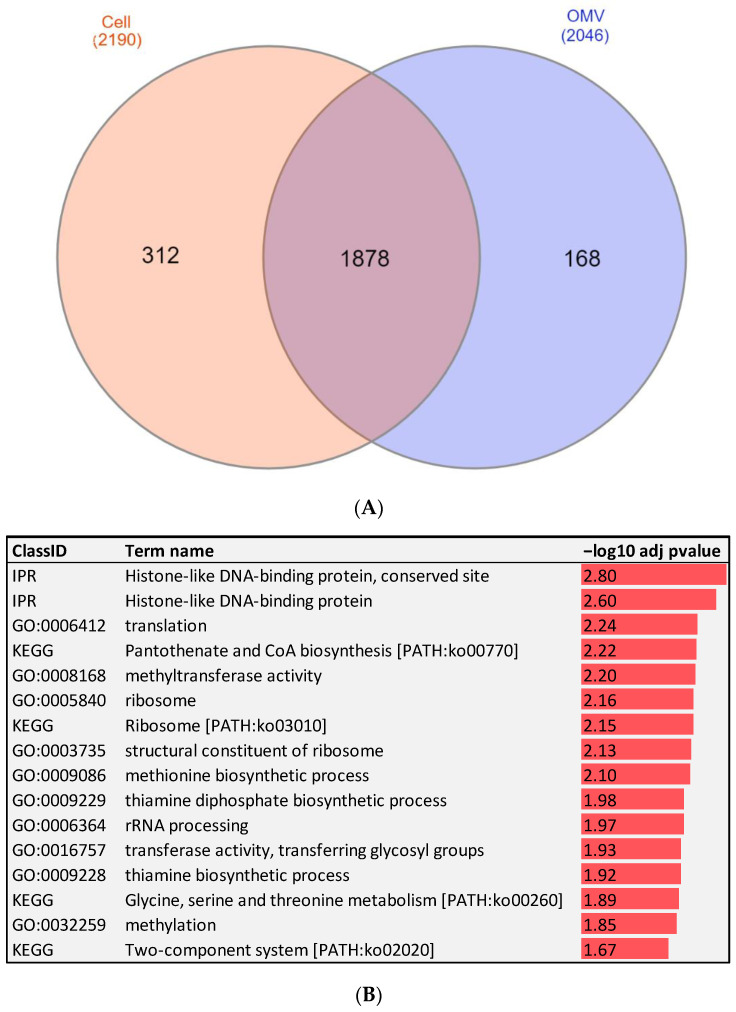
(**A**) Venn diagram showing OMV- and cell-unique and overlapping transcripts. (**B**) Enrichment analysis of abundant transcripts in the bacterial OMVs. Negative log10 adj *p*-values were plotted to show over-represented KEGG pathways, IPRs, and GO terms.

RNA-seq identified 2190 transcripts expressed in the whole cell and 2046 transcripts expressed in OMVs with an RPKM >0.45. The list of transcripts with their expression values is included in Supplementary File S1. Among these transcripts, 1878 were found in both OMVs and *Fp* whole cells, whereas 312 transcripts were uniquely expressed in the whole cell, and 168 were OMV-unique transcripts (Figure 2A). Several of those OMV-specific transcripts are essential for bacterial virulence. These transcripts included cold shock domain-containing protein (CSPs), threonine ammonia-lyase IlvA (threonine dehydratase), integration host factor (IHF) subunit beta, and co-chaperone GroES (Table 1). The bacterial CSPs function as regulators of the expression of stress resistance and virulence genes, thereby promoting host pathogenicity [16]. Threonine dehydratase is essential for pneumococcal virulence in mice [17], and IHF is a positive regulator of virulence gene expression in Gram-negative bacteria [18].

Transcripts, common to both OMVs and whole cells showed differential abundance. A total of 214 transcripts were more abundant in OMVs, whereas 1143 transcripts were more expressed in the whole cell (fold-change ≥2). Remarkably, ribosome-related transcripts were the dominant differentially abundant transcripts in the OMV compared to levels in the whole cell (Table 2).

To gain insights into the biological function of the cell- versus OMV-unique and most significantly enriched transcripts (fold-change ≥15), we performed gene enrichment analysis (Figure 2B). Abundant transcripts in the *Fp* whole cell were significantly enriched in energy production and conversion (Table 2). The list included NAD (P) FAD-dependent oxidoreductase, cytochrome-c cbb3-type subunit I, and 4Fe-4S dicluster domain-containing. Conversely for OMVs, the analysis showed an over-representation of genes mapped to four KEGG pathways, ribosome, pantothenate and CoA biosynthesis, glycine, serine and threonine metabolism, and two-component system. Furthermore, the analysis showed the enrichment of GO terms associated with methyltransferase activity, rRNA processing, structural constituent of ribosome, translation, methionine biosynthetic process, and thiamine diphosphate biosynthetic process. Some of the important OMV-enriched pathways and GO terms will be discussed in the following sections.

### 2.3. Ribosome

Remarkably, several ribosomal RNAs were among the most enriched in the OMVs compared to levels in the whole cell, suggesting the continuation of protein synthesis in the OMVs and most likely in the host cell following infection. The enriched ribosomal subunits included 30S ribosomal protein S16, 30S ribosomal protein S6, 30S ribosomal protein S18, 30S ribosomal protein S20, 50S ribosomal protein L33, 50S ribosomal protein L27, and 50S ribosomal protein L32. For instance, the transcript encoding 30S ribosomal protein S20 (FE46_RS09040) was ranked second among the most enriched in the OMVs (enrichment fold-change ~236). The enrichment of the ribosome components perhaps facilitates the production of more virulence factors necessary to hijack the host immune system. Previous reports showed that disrupting the optimal arrangement of the bacterial ribosomal components can lead to a loss of function and resistance to pathogens. Ribosome-targeting antibiotics lodge between the crucial ribosomal components to disrupt the synthesis of new proteins [19]. Our results suggest the bacterial ribosome as an interesting area of research to develop novel strategies that can contain the disease, such as the development of new ribosome-inhibiting antimicrobial drugs [20].

### 2.4. Two-Component System

Two-component signal transduction systems facilitate bacterial responses and adaptation to environmental or intracellular changes. Each two-component system includes a sensor histidine kinase protein, which receives a signal and transmits it to a response regulator. The latter transmits the signal to the target and induces changes in transcription [21]. Genes involved in the two-component system were uniquely represented in the OMVs. These genes included glycosyltransferase, LytTR family DNA-binding domain-containing protein, and the response regulator transcription factor (GerE).

Glycosyltransferase plays a crucial role in the assembly of peptidoglycan, which surrounds most bacteria and confers a stress-bearing shell [22]. Many Gram-negative bacteria interact with host cells by injecting proteins, such as glycosyltransferases, into infected host cells to post-translationally modify the structure and function of host proteins. Glycosyltransferases can modify protein substrates on arginine residues, which disrupts the normal functioning of the innate immune system [23]. Thus, glycosyltransferases have been suggested as great potential targets for anti-virulence compounds and the future of antibiotic discovery [22].

Furthermore, LytTR family DNA-binding domain-containing protein is a transcriptional regulator that controls the production of virulence factors in some bacterial pathogens [24], whereas GerE is a DNA-binding protein that works with σ^K^ to activate or repress gene expression. GerE binds to the promoter region of σ^K^-dependent genes; however, the mechanism by which GerE affects promoter activity is not known yet [25].

### 2.5. Pantothenate and CoA Biosynthesis

Pantothenate (vitamin B5) is the critical precursor for the biosynthesis of coenzyme A (CoA). CoA is a cofactor essential for the growth of pathogenic microorganisms and is implicated in various metabolic reactions, such as the tricarboxylic acid cycle, synthesis of phospholipids, and synthesis and degradation of fatty acids [26]. Genes involved in pantothenate and CoA biosynthesis were unique to the OMVs. These genes included the pantetheine-phosphate adenylyltransferase (PPAT) and biotin--acetyl-CoA-carboxylase ligase (BirA).

PPAT is essential in the CoA biosynthetic pathway to catalyze the reversible transfer of an adenylyl group from ATP to 4′-phosphopantetheine [27]. PPAT has been previously suggested as a candidate drug target to overcome antibiotic resistance [28]. The biotin protein ligase BirA represses the transcription of the biotin synthetic operon. BirA was identified as an essential component of a virulence-regulating pathway to allow bacterial adherence in a low biotin environment [29]. In addition, BirA regulates the expression of genes encoding heat and cold shock proteins perhaps to allow for bacterial survival under harsh environmental conditions [30].

### 2.6. Glycine, Serine, and Threonine Metabolism

Genes involved in glycine, serine, and threonine metabolism were enriched in the OMVs. These genes included aspartokinase and threonine ammonia-lyase IlvA (threonine dehydratase). Aspartate is an essential metabolite for bacterial virulence. Enzymes involved in aspartate metabolism, such as the aspartokinase, are suggested as promising targets for novel antibacterial compounds [31]. Moreover, threonine dehydratase *Streptococcus pneumoniae* mutants demonstrated in vitro decreased colonization, adhesion inability, and subsequently less virulence [17].

### 2.7. Genes with Enriched GO Terms

GO terms linked to ribosome, structural constituent of ribosome, translation, methionine biosynthetic process, thiamine diphosphate biosynthetic process, methylation, and methyltransferase activity were enriched in the OMVs.

Genes involved in the methionine biosynthetic process, such as aspartokinase and O-acetyl-L-homoserine sulfhydrolase, were significantly enriched in OMVs. Methionine is indispensable for many cellular processes, such as the initiation of protein synthesis and methylation of DNA, RNA, and proteins. The de novo methionine biosynthetic pathway is conserved in prokaryotes but absent in vertebrates, which makes methionine a potential antimicrobial target [32,33,34]. Previous studies demonstrated that the combined disruption of methionine biosynthesis and transport affected the growth and virulence of Salmonella [35].

Genes mapped to the thiamine diphosphate biosynthetic pathway encode hydroxymethylpyrimidine/phosphomethylpyrimidine kinase and HesA/MoeB/ThiF family protein. Thiamine (Vitamin B1) functions in the form of thiamine pyrophosphate (TPP) [36]. Bacteria synthesize the TPP or acquire it via the transportation of exogenous thiamine [37]. Deletion of the thiamine transporter (TT) operon in *Edwardsiella piscicida*, the causative agent of the edwardsiellosis disease in fish, resulted in attenuated pathogenicity, reduced host cell adhesion, and impaired abilities associated with motility [38].

Interestingly, GO terms associated with methylation and methyltransferase activity were also enriched in OMVs. Methyltransferase enzymes regulate the epigenetic landscape in prokaryotes and eukaryotes. Bacterial methyltransferases play an essential role in controlling the epigenetic information at the microbe level and in host–microbe interactions [39]. Methyltransferase activities target the host DNA and histone proteins, resulting in transcriptional changes in the host cell to enhance bacterial colonization [39]. Our results suggest that methyltransferases are promising targets to fight bacterial pathogenesis.

### 2.8. RNA-Seq of Pathogen on Day 5 Post-Infection of Fish from Resistant and Susceptible Genetic Lines

**Figure 3:**
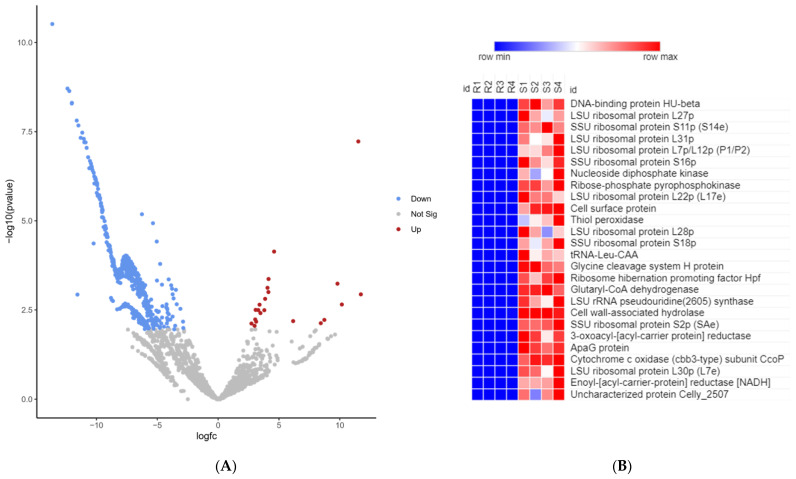
(**A**) Volcano plot showing DE bacterial transcripts between susceptible and resistant fish on day 5 post-infection (R/S). Upregulated transcripts in the resistant line are represented with red dots, whereas the downregulated transcripts are represented with blue dots (FDR ≤ 0.05). (**B**) Heat map showing the expression profile of the top DE transcripts (fold-change <−1000) between the susceptible and resistant fish on day 5 post-infection (R/S). The numbers at the top represent replicates per genetic line.

To identify potential bacterial transcript markers associated with disease susceptibility, we sought to investigate the variation in abundance of bacterial transcripts, in vivo, on day 5 following the infection of rainbow trout with *Fp*. For this purpose, we used fish collected from selectively bred resistant (ARS-*Fp*-R) and susceptible (ARS-*Fp*-S) genetic lines challenged with *Fp* as previously described in [40]. RNA-Seq from infected resistant and susceptible genetic lines yielded 372,100,715 raw sequence reads (average of 46,512,589 reads/sample). To identify differentially expressed (DE) bacterial transcripts, sequence reads were mapped to the pathogen’s reference genome [2]. A total of 4,870,725 reads (1.31%) were mapped to the *Fp* reference genome. Notably, 97.82% of the *Fp* mapped reads were generated from the susceptible genetic line, which is explained by a higher bacterial load as reported in the susceptible line compared to that in the resistant line [40]. Normalized gene expression was used to account for differences across samples by converting raw count data to RPKM values. A total of 576 bacterial transcripts were DE with a false discovery rate (FDR) <0.05 and a minimum fold-change value ≥2 or ≤−2 (Figure 3A,B and Supplementary File S2). Notably, 87 OMV-enriched transcripts were among the DE transcripts between the two genetic lines. Most of the DE bacterial transcripts (96.4%) were downregulated in the resistant line. More information about DE transcripts is given in Supplementary File S2.

**Figure 4:**
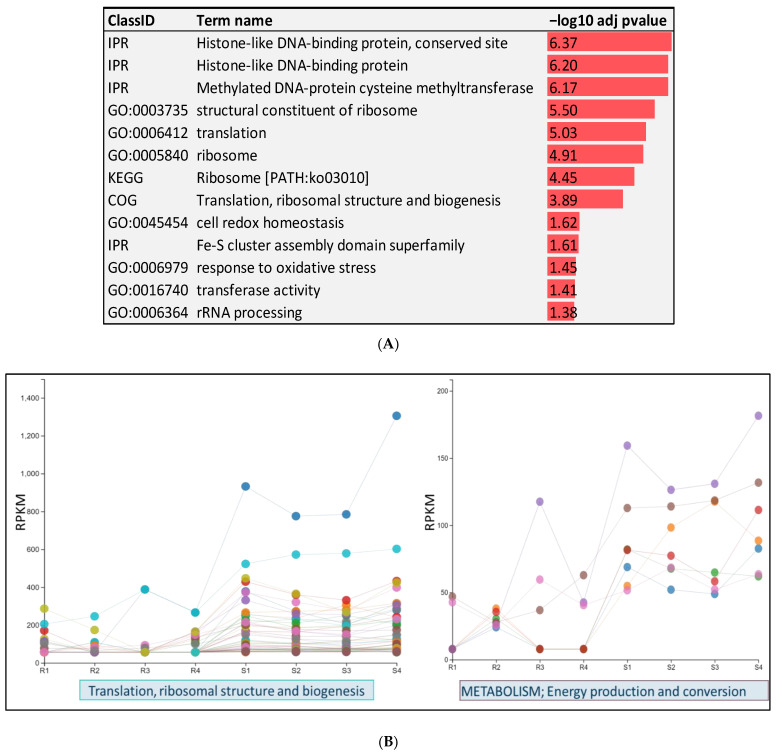
(**A**) Enrichment analysis of DE bacterial transcripts in susceptible and resistant fish on day 5 post-infection. Negative log10 adj *p*-values were plotted to show over-represented KEGG pathways, IPRs, and GO terms. (**B**) Line chart of the normalized expression values of genes involved in the translation machinery (**left** panel) and energy production/conversion (**right** panel) in the resistant and susceptible genetic lines. R1–R4 and S1–S4 represent replicates of the resistant and susceptible genetic lines. Each color represents one gene.

In total, 555 bacterial transcripts were downregulated in resistant fish on day 5 post-infection. Functional enrichment analysis was conducted to gain insights into the biological functions of the downregulated transcripts (Figure 4A). Transcripts encoding Fe-S cluster assembly ATPase, FeS assembly SUF system protein, Ferredoxin, Ferredoxin--NADP reductase, ferritin-like domain-containing protein, and nitrogen fixation were downregulated in *Fp* when the resistant fish genetic line was infected. Iron-sulfur clusters have a primary role in electron transfer and act as “molecular switches” for gene regulation [41]. Iron is a necessity for virulence in most pathogenic bacteria, and heme/iron transport is a virulence determinant of *Fp* [42,43]. Moreover, bacteria require nitrogen to synthesize core cell constituents, such as purines, pyrimidines, and amino sugars [44]. Genes involved in DNA replication and the synthesis of RNA primer were significantly underrepresented in the resistant fish. Consistently, genes promoting ribosome structure and biogenesis or enhancing the efficiency of translation machinery were among the most downregulated in resistant fish (Figure 3B and Figure 4B). Twenty-seven genes were mapped to the ribosome KEGG pathway. Ribosome and translation-related genes were also identified among the most over-represented genes in OMVs (Table 2). Our results provide initial evidence for the potential crucial role of the bacterial ribosome and translation machinery in hijacking the host immune response and rendering the fish susceptible to disease.

In resistant fish, we noticed an under-representation of bacterial genes implicated in the response to oxidative stress, such as thiol peroxidase, glutathione peroxidase, peptide-methionine (R)-S-oxide reductase, and alkyl hydroperoxide reductase. This suggests that bacteria in the resistant fish are more vulnerable to oxidative stress, as the production of a complex mixture of oxidants is a major host defense mechanism against invading pathogens [45]. In addition, several bacterial genes involved in DNA methylation were under-represented in resistant fish. The list includes methylated-DNA--protein-cysteine methyltransferase, 23S rRNA (guanosine (2251)-2′-O)-methyltransferase, and tRNA (guanosine (18)-2′-O)-methyltransferase. Methylation is one of the mechanisms by which pathogens can control host functions [46]. Besides their role in microbial epigenetic regulation, bacterial methyltransferase enzymes have a crucial role in host–microbe interactions [39].

Histone-like DNA-binding proteins, such as integration host factor (IHF) alpha/beta and DNA-binding protein HU-beta, were under-represented in resistant fish. IHF alpha/beta binds the minor groove of DNA to induce a large bend, which stabilizes distinct DNA conformations essential for bacterial recombination, transposition, replication, and transcription. IHF contributes to bacterial survival in highly competitive environments [47]. Notably, DNA-binding protein HU-beta was the most downregulated bacterial transcript in resistant fish (fold-change −12,667.9) (Figure 3B). DNA-binding protein HU-beta wraps the DNA to stabilize it under extreme environmental conditions [48].

Remarkably, *Fp* genes with hydrolase activity were significantly downregulated in resistant fish on day 5 following infection (Table 3). These *Fp* genes included CWH, GTP cyclohydrolase I, isopentenyl-diphosphate delta-isomerase, NUDIX hydrolase, and Rhs-family protein. *Fp* CWH was the most downregulated hydrolase (fold-change −1204.8) in the resistant fish (Supplementary File S2). GTP cyclohydrolase I is the first enzyme of the de novo tetrahydrofolate biosynthetic pathway in bacteria [49], whereas isopentenyl diphosphate (IPP) isomerase catalyzes the conversion of IPP to dimethylallyl diphosphate (DMAPP), an essential step in the isoprenoid biosynthetic pathway [50]. Several in vivo and in vitro studies linked isoprenoid synthesis to the intracellular survival of pathogenic bacteria. Consequently, isoprenoid synthesis was suggested as a target to inhibit bacterial growth [51]. Furthermore, nudix proteins catalyze the hydrolysis of pyrophosphate bonds and have a demonstrated role in bacterial fitness and virulence. Mutants of *Pseudomonas aeruginosa* devoid of individual nudix hydrolases were more sensitive to killing by oxidative stress/H_2_O_2_ and showed less virulence [52]. Moreover, Rhs proteins play a crucial role in the interaction between bacteria and host cell. The Rhs protein has anti-phagocytosis activities and facilitates bacterial adhesion and invasion abilities. Rhs mutants showed a significant decrease in bacterial ability for multiplication in vivo [53]. Taken together, this study helps in understanding the mechanism governing *Fp* pathogenesis and establishing a foundation for further research.

### 2.9. Cell Wall Hydrolase (CWH)

CWH, a hydrolytic enzyme, was the most abundant transcript in OMVs (RPKM = 55,688) (Supplementary File S1). Cell wall hydrolases are enzymes involved in cell lysis during bacterial cell division [54]. *Fp* contains several other enzymes besides cell wall hydrolase involved in host invasion. Genome-wide prediction in *Fp* determined another hydrolytic enzyme similar to an elastinolytic enzyme, which resembles an enzyme present in pathogenic bacteria, such as *Pseudomonas*, *Vibrio*, and *Leptospiria,* and these enzymes enable invasion, tissue necrosis, and increased vascular permeability in the host [55]. However, the role of the hydrolytic enzymes, specifically cell wall hydrolase, in pathogenesis has not been explained clearly yet in most bacteria. The enrichment of the CWH gene in OMVs compared to that in the transcriptome of the whole bacterial cells (3.56-fold) and CWH downregulation in resistant fish on day 5 following infection (Table 3) might suggest a specific function of this gene in lysing the bacterial or host cell during the bacterial–host interaction. Some studies indicated a cell wall hydrolase role in bacterial growth and division by controlling the degradation of the peptidoglycan layer in bacteria, and that are thus referred to explicitly as peptidoglycan hydrolases [56,57]. A recent study on Gram-positive bacteria detected proteomic enrichment of four cell wall hydrolases in OMVs and suggested the involvement of CWH in the formation of the OMV [58].

### 2.10. CWH Is Conserved among Many Strains of Fp

The CWH transcript was conserved in 51 out of 64 studied strains of *Fp* with a sequence identity ≥99% and query coverage >99%. In the genome of *Fp* CSF 259-93, the strain used for sequencing in this study, there were four copies of this gene [2]. We investigated CWH conservation in seven highly virulent versus three less virulent strains of *Fp.* The highly virulent strains were *Fp* G10, *Fp* G101, JIP02-86, *Fp* S-S6, OSU THCO2-90, JIP 08/99, JIP 16/00, 950106-1, and *Fp* G3 [55,59,60,61], and the less virulent strains were CR, *Fp* GIW08, and NCIMB 1947 [59,60,62]. The CWH transcript was conserved in six (highly virulent) strains with 100% sequence identity and three (low virulence strains) (Supplementary File S3). As these enzymes are primarily involved in cell lysis during bacterial cell division, cell wall synthesis, development [63], and OMV formation [58], they might be crucial for bacterial survivability, which might explain the evolutionary conservation of this gene in a vast number of *Fp* strains.

### 2.11. Genetic Manipulation of the Cell Wall Hydrolase Gene Failed in Fp

To investigate the role of the CWH in the pathogenesis of *Fp*, we tried to delete this gene in *Fp*. For this, we used a pyt313 suicide vector carrying the sacB, Amp^r^ (Em^r^) gene generated by Barbier et al. [64]. We incorporated an insert in this plasmid and then subjected it to conjugation. After cell plating, patches of pale-yellow colonies were observed, and those patches were screened via PCR using Srn primers, and an expected 1.1 kb band was observed upon running the gel electrophoresis. However, no bacterial growth was observed in erythromycin tryptone yeast extract agar plates. This might be due to difficulties in transferring the plasmid to *Fp* based on the strain used, which might be due to restriction enzymes produced by the bacteria used in our study.

## 3. Conclusions

The current study characterized and functionally annotated the OMV and *Fp* whole-cell transcriptomes and investigated variation in the bacterial transcript abundance on day 5 following infection of resistant and susceptible fish with *Fp*. Interestingly, ribosome-related transcripts were highly enriched in the OMV and susceptible fish indicating an essential role for the bacterial translation machinery in pathogenesis. The study revealed a potential role for the histone-like DNA-binding proteins and bacterial methyltransferases in the host–microbe interaction. The CWH was the most abundant transcript in OMVs and among the top upregulated transcripts in susceptible fish on day 5 following infection, suggesting this gene’s role in lysing the bacterial cell and host cell, forming and merging OMV with host cells during the host–microbe interaction. The CWH was subjected to gene silencing; however, it could not be accomplished in the *Fp* strain CSF-259-93. This might be because the restriction enzymes produced by *Fp* cause difficulties in the conjugal transfer of a plasmid. Further molecular characterization should be performed to understand the function of CWH in mediating cell lysis in the host.

## 4. Materials and Methods

### 4.1. Bacterial Strain and Growth Condition

*Fp* strain CSF-259-93, kindly provided by Dr. Gregory Wiens, NCCCWA/ARS/USDA, was used in our study. A frozen stock culture of *Fp* was cultured on tryptone yeast extract agar with an agar percentage of 1.5%, with a 0.02% beef extract [65] plate, and the plate was incubated at 15 °C for one week. *Fp* colonies were then transferred to tryptone yeast extract broth, and absorbance (525 nm wavelength) was measured every day for 2 weeks to determine the log phase of the cultures. Measurement of *Fp* density by measuring the OD in the broth culture indicated that the log phase existed between days 5 and 11 (Supplementary File S4), and day 8 was used for OMV isolation. Tryptone yeast extract broth culture without *Fp* was used as a negative control.

### 4.2. Isolation of OMVs

*Fp* broth culture was used to increase the bacterial mass, and OMVs were isolated from *Fp* broth culture on day 8 of bacterial growth. OMVs were isolated from bacterial cells for downstream RNA sequencing and the prediction of bacterial genes. The experimental design is shown in Supplementary File S4. A loopful of culture was subcultured on a plate on day 7 of bacterial growth to ensure that the broth was contamination-free. For OMV isolation, broth culture from a flask was distributed into several 50 mL tubes. Each tube was centrifuged at 2800× *g* for 1 h at 4 °C to pellet the bacterial cells. The supernatant was collected and filtered through a 250 mL sterile 0.22 µm PES membrane filter (EMD Millipore Corporation, Billerica, MA, USA) to filter any remaining bacterial cells. The filtrate was then subjected to ultracentrifugation (Beckman Coulter Optima L-90K, 40 Ti rotor) for 3 h at 40,000 rpm (285,000× *g*) at 4 °C to pellet the OMVs. The OMV pellet was then washed with phosphate-buffered saline (PBS) buffer and again subjected to ultracentrifugation for 2 h at 40,000 rpm at 4 °C to re-pellet the OMVs. The OMV pellet was then resuspended in nuclease-free (NF) water and stored at −20 °C. The protein concentration of the OMVs was quantified using a BCA Protein assay kit (Thermo Fisher Scientific, Waltham, MA, USA). To ensure that the suspension containing OMVs was free from bacteria, 30 µL of the suspension was cultured on a tryptone yeast extract agar plate and incubated for 10 days at 18 degrees Celsius.

### 4.3. Transmission Electron Microscopy (TEM) of OMVs

Transmission electron microscopy (TEM) was performed on *Fp* OMVs and *Fp* bacterial cell samples. For *Fp* bacterial cells, a single colony from a tryptone yeast extract agar plate was suspended in nuclease-free water. For *Fp* OMVs, TEM was performed using OMV suspensions. Using a dropper, 2 drops of OMVs suspended in nuclease-free water were deposited on carbon-coated grids and incubated for 2 min. The excess sample was removed from the grid using blotting paper. Nuclease-free water and tryptone yeast extract broth were used as a negative control. All samples (*Fp* whole cells and *Fp* OMVs) were subjected to negative staining using uranyl acetate. Briefly, samples were deposited on TEM carbon coated grids (80 mesh square grid, EMS, TED PELLA, Inc., Redding, CA, USA) and incubated for 2 min. Samples were blotted dry, and grids were washed with sterile deionized water three times (30 s each) to remove the salt buffer. Before the samples were stained, excess water from grids was removed with blotting paper. To stain the samples, 5 µL of 1% uranyl acetate was added onto the grid and incubated for about 1 min. The stain was then washed with sterile deionized water and dried, and finally, the grids were observed under a Hitachi H-7650-II instrument (Schaumburg, IL, USA) for TEM.

### 4.4. RNA Extraction, Library Preparation, and Sequencing

RNA was extracted from *Fp* colonies isolated from a tryptone yeast extract agar plate for whole *Fp* cells and from OMVs isolated from *Fp* broth culture using TriZol reagent (Invitrogen, Carlsbad, CA, USA). OMV RNA, RNase-treated, and untreated OMV RNA samples were run based on agarose gel electrophoresis for confirmation. For RNAse treatment, 4 µL of the RNA samples was treated with RNase (Invitrogen RNase Cocktail Enzyme mix) (Thermo Fisher Scientific, Waltham, MA, USA) (2 µg/µL) and incubated in a water bath at 37 °C for 30 min. RNA samples were stored at −80 °C until subjected to further processing.

For library preparation and sequencing, samples were sent to BGI Genomics (Cambridge, MA, USA). The library preparation was performed using a Trio RNA-seq kit (NuGEN, San Carlos, CA, USA) according to the manufacturer’s recommendations. Briefly, an rRNA depletion step was performed for mRNA enrichment. The enriched mRNA was then fragmented into small pieces using a fragmentation buffer and purified using a QiaQuick PCR extraction kit, the solution was resuspended in EB buffer, and cDNA was then subjected to end repair and poly (A) tail addition. The fragments were then connected with adaptors. The library was then purified using a MiniElute PCR Purification kit before PCR amplification. The libraries were amplified via PCR, and then, the yield was quantified. Sequencing was performed using 100 bp-paired end sequencing on an Illumina Miseq.

### 4.5. Data Processing and Functional Prediction of Transcripts

After sequencing, raw reads were filtered, including removing adaptor sequences, contamination, and low-quality reads. A total of 60,352,578 *Fp* RNA clean reads and 55,722,742 OMVs clean reads were subjected to downstream analysis using the QIAGEN CLC Genomics workbench (version 12.0.3, CLC bio, Aarhus, Denmark; http://www.clcbio.com/products/clc-genomics-workbench/, accessed on 13 March 2019). Five base pairs from the forward ends and 5 bp from the reverse ends were trimmed to remove low-quality nucleotides. The trimmed reads were then mapped to the *Fp* CSF-259-93 reference genome, NCBI accession GCF_000739395.1 [2]. Mapping parameters included mismatch cost = 2, insertion/deletion cost = 3, minimum length fraction = 0.9, and similarity fraction = 0.9. To determine the abundance of genes, the expression values of transcripts were calculated in terms of reads per kilobase per million (RPKM).

### 4.6. Bacterial Challenge of BCWD-Resistant and BCWD-Susceptible Fish Population

Tissue samples from resistant and susceptible rainbow trout genetic lines were obtained from the USDA/NCCCWA (Provided by Dr. Gregory D Wiens). The genetic lines were developed by the USDA-NCCCWA via a family-based selection method as previously described [40,66]. In brief, within the genetic lines, single-sire × single-dam matings were established between 3-year-old dams and 1-year-old sires (neo-males). To enhance the disease-resistance phenotype, the resistant line dams and sires had undergone three and four generations of BCWD selection. In contrast, the susceptible line parents had undergone one generation of selection to allow for a higher susceptibility to infection. Significant differences in susceptibility to *Fp* were previously observed between the resistant and susceptible genetic lines [40,67].

As previously described by Marancik et al. [40], fish from resistant and susceptible genetic lines were challenged with *Fp* (49 days post-hatch). In brief, fifty fish from each genetic line were randomly allocated to two tanks (2.4 L min^−1^ of 12.5 ± 0.1 °C flow-through spring water supply), and then, fish were intraperitoneally injected with 4.2 × 10^6^ CFU fish^−1^ *Fp* suspended in 10 μL PBS. Similarly, two fish tanks were injected with PBS for each genetic line as a non-infected control. The fish survival was monitored for 21 days. Five fish from each tank were sampled on day 5 post-infection. All fish used in this study were certified as infection-free before injection with *Fp.*

### 4.7. Sequencing and Differential Gene Expression Analysis of BCWD-Resistant and BCWD-Susceptible Fish

RNA was isolated from the whole fish (1.1 g fry) using TriZol (Invitrogen, Carlsbad, CA, USA). Quantity and quality assessments were performed as previously described [68]. To eliminate potential DNA contamination, RNA samples were treated with DNAase I (Fisher BioReagents, Hudson, NH, USA). Equal amounts of RNA were pooled from 2 fish, and 4 pools (2 samples/pool) from each resistant and susceptible genetic line were sequenced (i.e., a total of 8 libraries). RNA was sequenced at RealSeq Biosciences, Inc. (Santa Cruz, CA, USA). The Zymo Ribofree library prep kit (Irvine, CA, USA), targeting the host and bacterial RNAs, was used during the rRNA-depleted library preparation.

Raw RNA-Seq datasets were submitted to the NCBI Short Read Archive under BioProject ID PRJNA259860. As previously described, raw sequence reads generated from each genetic line were subjected to a quality check and trimming [68]. High-quality reads were mapped to the *Fp* reference genome [2] using a CLC genomics workbench to identify DE transcripts. Mismatch cost = 2, insertion/deletion cost = 3, minimum length fraction = 0.9, and similarity fraction = 0.9 were allowed during mapping. Unmapped reads, including rainbow trout reads, were filtered out. Supplementary File S4 shows sequence read counts and mapping statistics.

The expression value of each transcript was calculated in terms of RPKM, and then, the EDGE test was used to identify DE transcripts between resistant and susceptible genetic lines (*p*-value FDR < 0.05, fold change cutoff ±2). Gene set enrichment analysis was performed using FUNAGE-Pro with an adj *p*-value < 0.05 [69]. Supplementary File S4 shows the principal component analysis of eight RNA-seq datasets generated from selectively bred, resistant- and susceptible-line rainbow trout on day 5 post-infection and the validation of RNA-Seq data via qPCR for selected genes.

### 4.8. Conservation of CWH Transcripts

To determine the conservation of CWH transcripts, genome sequences were downloaded from 64 strains of *Fp* from NCBI https://www.ncbi.nlm.nih.gov/genome/browse/#!/prokaryotes/1589/, accessed on 7 May 2020 (Supplementary File S3). CWH transcripts were blasted against all 64 *Fp* strains using a local BLAST in Bioedit [70]. The conservation of transcripts in more and less virulent strains was determined by blasting the transcript with their genomes, respectively. The cutoff value includes query coverage >99% and identity ≥99% of the matching sequencing.

### 4.9. Genetic Manipulation of CWH Gene

#### Construction of the CWH Deletion Mutant

Efforts to genetically manipulate CWH were conducted using a modified method of Barbier et al. [64]. For the effort to delete CWH from *F. pyschrophilum* (CSF-259-93), a ~3 Kbp fragment upstream of 2995 bp and downstream of 2947 bp was used to amplify the transcripts associated with the cell wall hydrolase gene using Green Taq polymerase and primers CWHus (introducing a BamHI site in the forward primer and SalI in the reverse primer) and CWHds (introducing a PstI sit in both forward and reverse primers). The CWHus fragment was then digested with BamHI and SalI and ligated into the suicide vector pYT313 (kindly provided by Dr. Mark J. McBride). The plasmid was digested with the same enzymes. To the vector, rSAP (shrimp alkaline phosphatase) was added to prevent recircularization during ligation. After ligation, the transformation was performed using competent cells of *E. coli*, strain S17-1 (λ-pir). The transformed cells were PCR-screened to confirm the transformation of the insert into the plasmid. The colonies were then cultured in LB broth with ampicillin in it. The plasmid containing the CWHus insert was then subjected to purification using a QIAprep Spin Miniprep Kit (Qiagen, Germantown, MD, USA). The procedure was repeated with the CWHds primer, which had been ligated with the PstI enzyme, and the vector PYT313 incorporated with the CWHus insert had also been digested with the same PstI enzyme. Since we used the same restriction enzyme at both ends of CWHds, to ensure proper orientation of the insert, we designed the primer upstream and downstream of CWHds, and colony screening of the transformants was performed using screening (CWHscrn) primers. A band size of approximately 1.1 kb was observed upon running gel electrophoresis. Plasmid incorporated with our insert CWHus and CWHds was then transferred to *Fp* CSF-259-93 via conjugation, and the colonies having the plasmid incorporated into the chromosome through recombination were selected by screening for erythromycin resistance colonies. Resistant colonies were streaked on tryptone yeast extract agar.

### 4.10. Conjugative Transfer of Plasmid into F. psychrophilum

Conjugation was used to transfer the plasmid from *E. coli* strain S17-1 (λ-pir) into *Fp* strains. Briefly, *E. coli* strains were grown overnight in 5 mL LB broth with shaking at 37 °C. Similarly, *Fp* strains were also grown at 15 °C for 4 days in 5 mL tryptone yeast extract broth. Cells from *E. coli* and *Fp* cells were collected via centrifugation at ~10,000 rpm for 25 min and washed twice with 1 mL LB broth for *E. coli* cells and tryptone yeast extract broth for *Fp* cells. *E. coli* cells were resuspended in 500 µL LB broth, and *Fp* cells were resuspended in 500 µL of tryptone yeast extract broth. Both suspensions were mixed, cells were then spotted on tryptone yeast extract agar using a micropipette, and the plates were incubated at 17 °C for 4 days. After incubation, cells were removed from the plate using a scrapper and suspended in 2 mL tryptone yeast extract broth. From the suspension, 100 µL of the aliquots was spread on tryptone yeast extract agar containing erythromycin (10 ug/mL). The plates were then incubated for 7 days at 17 °C. The colonies were then PCR-screened using the CWH Scrn primer (upstream and downstream of CWH DS region). An isolated colony was inoculated in tryptone yeast extract broth without erythromycin, and the broth was incubated at 17 °C to allow for the loss of an integrated plasmid. Recombinant plasmids were screened by culturing on 50 g/L sucrose-containing tryptone yeast extract agar, and the plate was incubated at 17 °C. All primers used in this study are included in Table 4.

## Figures and Tables

**Table 1:** OMV-versus whole cell-unique mRNA transcripts. Several OMV-unique transcripts are essential for bacterial virulence.

Feature ID	Gene Description	Differential Abundance (OMVs/*Fp* Whole Cell)
FE46_RS03875	Hypothetical protein IA03_02225	258.63
FE46_RS01465	Cold shock domain-containing protein	236.83
FE46_RS04325	DUF3820 family protein	40.87
FE46_RS03890	Co-chaperone GroES	28.51
FE46_RS08245	Putative membrane spanning protein	25.95
FE46_RS05500	Integration host factor subunit beta	23.18
FE46_RS05600	CDP-alcohol phosphatidyltransferase family protein	18.81
FE46_RS02350	Threonine ammonia-lyase IlvA	18.17
FE46_RS04330	OsmC family protein	15.54
FE46_RS02255	Cadmium-translocating P-type ATPase	−223.61
FE46_RS12900	Leucine-rich repeat protein	−193.17
FE46_RS10250	Family transcriptional regulator	−186.12
FE46_RS11155	Ketoacyl-ACP synthase III	−127.88
FE46_RS02245	Acetyl-hydrolase transferase family	−117.56
FE46_RS12880	Leucine-rich repeat protein	−100.47
FE46_RS12920	Leucine-rich repeat protein	−82.91
FE46_RS12885	Leucine-rich repeat protein	−79.44
FE46_RS03485	Division cell wall cluster transcriptional repressor	−60.93
FE46_RS03530	UDP-N-acetylmuramate--L-alanine ligase	−59.90
FE46_RS11460	NADH-quinone oxidoreductase subunit B	−53.92
FE46_RS04540	DUF2147 domain-containing	−51.66

**Table 2:** Differentially abundant transcripts between the *Fp* whole cells and OMVs. Pervasive enrichment of transcripts involved in translation, ribosomal structure, and biogenesis was observed in OMVs. In contrast, energy production and conversion genes were enriched in the whole cell.

Feature ID	Gene Description	Differential Abundance (OMVs/*Fp* Whole Cell)
FE46_RS03770	HU family DNA-binding protein	250.39
FE46_RS09040	30S ribosomal protein S20	236.37
FE46_RS03985	KTSC domain-containing protein	124.10
FE46_RS03320	Family outer membrane	61.13
FE46_RS09965	Type B 50S ribosomal L31	43.71
FE46_RS09215	50S ribosomal L33	37.49
FE46_RS12230	50S ribosomal L32	33.37
FE46_RS01825	30S ribosomal S16	30.62
FE46_RS01795	Inorganic pyrophosphatase	29.61
FE46_RS12455	Copper resistance	28.99
FE46_RS11385	50S ribosomal L27	23.06
FE46_RS05880	3,4-Dihydroxy-2-butanone-4-phosphate synthase	22.28
FE46_RS04555	30S ribosomal S6	21.17
FE46_RS04560	30S ribosomal S18	19.11
FE46_RS02470	Ribosome assembly cofactor	16.53
FE46_RS05480	NAD(P) FAD-dependent oxidoreductase	−158.95
FE46_RS10070	Cytochrome-c cbb3-type subunit I	−149.66
FE46_RS08120	4Fe-4S dicluster domain-containing	−125.13
FE46_RS10110	Aconitate hydratase	−59.32
FE46_RS10085	Cytochrome c oxidase accessory	−56.74
FE46_RS01775	Electron transfer flavo subunit alpha family	−40.12
FE46_RS12380	2-Oxoglutarate dehydrogenase complex dihydrolipoyllysine-residue succinyltransferase	−37.85
FE46_RS01970	4Fe-4S dicluster domain-containing	−33.28
FE46_RS12375	2-Oxoglutarate dehydrogenase E1 component	−26.94
FE46_RS02175	L-glutamate gamma-semialdehyde dehydrogenase	−25.98
FE46_RS06595	Succinate--ligase subunit alpha	−24.98
FE46_RS01780	Electron transfer flavo subunit beta family	−24.12
FE46_RS05145	Dihydrolipoyl dehydrogenase	−23.63
FE46_RS08480	FAD-binding	−22.90
FE46_RS05800	Class II fumarate hydratase	−22.35
FE46_RS11950	Aldehyde dehydrogenase family	−21.72
FE46_RS00480	Succinate dehydrogenase/fumarate reductase iron-sulfur subunit	−17.80

**Table 3:** Differentially expressed *F. psychrophilum* transcripts, with hydrolase activities, in susceptible and resistant rainbow trout fish on day 5 post-infection.

Feature ID	Fold Change (R/S)	*p*-Value FDR	Gene Description
FE46_RS09020	−1204.81	0.002	Cell wall-associated hydrolase
FE46_RS07385	−348.46	0.003	TIGR00730 family Rossman fold protein
FE46_RS11855	−306.96	0.003	Rhs-family protein
FE46_RS04860	−291.40	0.003	Isopentenyl-diphosphate delta-isomerase
FE46_RS08365	−288.91	0.003	GTP cyclohydrolase
FE46_RS08015	−116.46	0.022	NUDIX domain-containing protein
FE46_RS08160	−84.13	0.006	GTP cyclohydrolase I
FE46_RS11250	−8.59	0.018	Cell wall-associated hydrolase

**Table 4:** Primers used in this study.

Restriction Enzyme	Primers	Tm (Degree C)
CWH1us(Bam)	5′ actactGGATCCTAAAAGACAAAATATGCTAGATGG 3′	61
CWH1us(Sal)	3′ actactGTCGACTTATGTACACACTTTTCCCGAG 5′	62
CWH1ds(Pst)	5′ actactCTGCAGTTTCTAGCCATTAGCCATTAG 3′	60
CWH1ds(Pst)	3′ actactCTGCAGTTATCAAATCCGTGTCATCTG 5′	60
CWH1ko(scrn)	5′ GAATTTAGAAATATTTATGAAGAAAC 3′	60
CWH1ko(scrn)	3′ TCTCGTAGCTCAGCTGGTTAG 5′	61

## Data Availability

The waw RNA-Req data that support the findings of this study are openly available in the NCBI Short Read Archive under BioProject ID PRJNA259860.

## Supplementary Materials

The following supporting information can be downloaded at: https://www.mdpi.com/article/10.3390/pathogens12030436/s1, Supplementary File S1: Transcripts identified in the *Fp* whole cells and OMVs; Supplementary File S2: Differentially expressed bacterial transcripts in susceptible and resistant fish on day 5 post-infection (R/S); Supplementary File S3: CWH conservation in different *Fp* strains; Supplementary File S4: Method supporting materials.
