# Supplementary material for: Dual RNA-Seq of Flavobacterium psychrophilum and Its Outer Membrane Vesicles Distinguishes Genes Associated with Susceptibility to Bacterial Cold-Water Disease in Rainbow Trout (Oncorhynchus mykiss)
Source: Pathogens. 2023 Mar 10;12(3):436. doi: 10.3390/pathogens12030436 (PMC10057207; doi:10.3390/pathogens12030436)
Supplement: Supplementary file 1 [file pathogens-12-00436-s001.zip › Supplementary file S4.pdf]

Supplementary file S4.

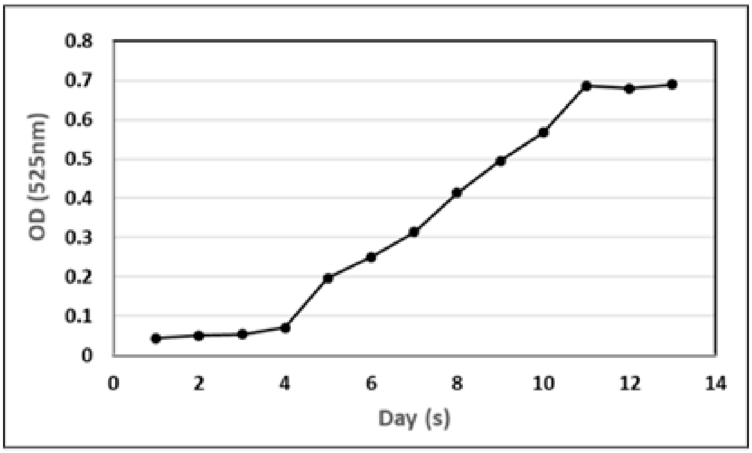

Measurement of *Fp* density by measuring OD in broth culture, the log-phase was observed between days 5 and 11, and day 8 was used for OMV isolation.

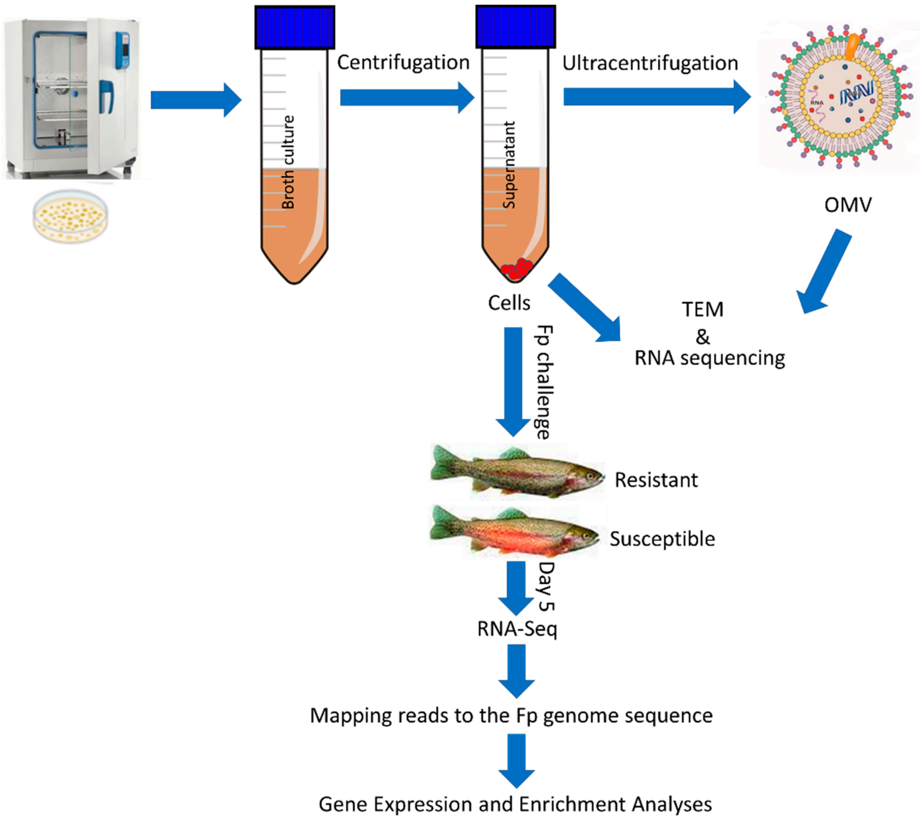

Overview of the experimental design to separate the Fp and OMVs as well as identifying markers associated with disease susceptibility. Fp and OMVs were visualized by the TEM. Whole-cell and OMV-packaged transcripts were sequenced on an Illumina MiSeq platform. Fish from BCWD-resistant and -susceptible genetic lines were intraperitoneally injected with *Fp*. Fish were collected on day 5 post-infection and processed for sequencing. Sequencing reads were mapped to the *Fp* genome sequence followed by gene expression and enrichment analyses.

Table showing sequence read counts and mapping statistics

	**Resistant genetic line**				**Susceptible genetic line**			
	R1	R2	R3	R4	S1	S2	S3	S4
Raw reads	47,817,215	58,217,219	52,507,328	33,920,459	43,924,736	67,089,323	43,120,037	25,504,398
Trimmed reads	47,798,832	58,179,320	52,494,547	33,908,307	43,910,762	67,062,878	43,107,809	25,496,664
Reads mapped (%)	22,634 (0.05)	44,416 (0.08)	15,238 (0.03)	24,096 (0.07)	1,026,439 (2.34)	1,939,010 (2.89)	1,317,621 (3.06)	481,271 (1.89)
Reads not mapped	47,776,198	58,134,904	52,479,309	33,884,211	42,884,323	65,123,868	41,790,188	25,015,393

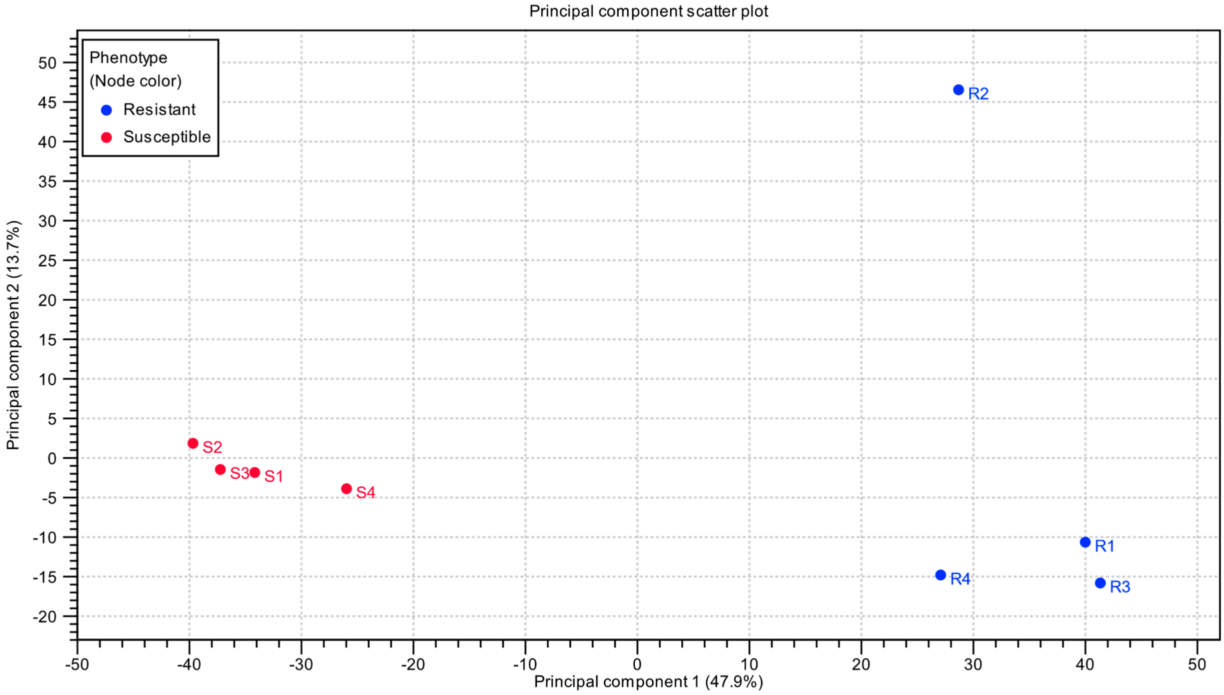

Principal component analysis of eight RNA-seq datasets generated from selectively bred, resistant- and susceptible-line rainbow trout on day 5 post-infection. RNA-seq datasets of each genetic line

are represented in color-coded dots. PCA1 accounts for 47.9% of the variation whereas PCA2 accounts for 13.7%.

Table: Validation of RNA-Seq data by qPCR. qPCR quantification revealed consistency with the RNA-Seq (R = 0.91; p-value 0.002).

**Feature ID**	**Gene description**	**qPCR**		**RNA-Seq**	
		**Fold change (R/S)**	***p*-value**	**Fold change (R/S)**	**FDR *p*-value correction**
FE46_RS12920	leucine-rich repeat protein	-99.26	3.68E-03	-670.98	6.26E-04
FE46_RS04860	isopentenyl-diphosphate Delta-isomerase	-105.60	1.83E-05	-291.40	2.90E-03
FE46_RS05480	Ferredoxin--NADP reductase	-20.30	2.92E-02	-314.15	2.98E-03
FE46_RS05500	Integration host factor subunit beta	-185.63	4.58E-06	-955.04	1.32E-04
FE46_RS09020	Cell wall-associated hydrolase	-39.60	4.96E-02	-1204.81	1.52E-03
FE46_RS09040	30S ribosomal protein S20	-25.99	5.06E-03	-302.23	4.21E-03
FE46_RS10250	PspC family transcriptional regulator	-54.43	4.61E-03	-788.16	2.12E-04
FE46_RS11385	50S ribosomal L27	-404.50	6.48E-05	-5325.84	1.99E-06
